# Leveraging open data to reconstruct the Singapore Housing Index and other building-level markers of socioeconomic status for health services research

**DOI:** 10.1186/s12939-021-01554-8

**Published:** 2021-10-03

**Authors:** Daniel Yan Zheng Lim, Ting Hway Wong, Mengling Feng, Marcus Eng Hock Ong, Andrew Fu Wah Ho

**Affiliations:** 1 Health Services Research Unit, Medical Board, Singapore General Hospital, Outram Road, Singapore, 169608 Singapore; 2 Department of General Surgery, Singapore General Hospital, Singapore, Singapore; 3 Pre-hospital and Emergency Research Centre, Health Services and Systems Research, Duke-NUS Medical School, Singapore, Singapore; 4 Saw Swee Hock School of Public Health, National University of Singapore, Singapore, Singapore; 5 Institute of Data Science, National University of Singapore, Singapore, Singapore; 6 Department of Emergency Medicine, Singapore General Hospital, Singapore, Singapore

**Keywords:** Socioeconomic status, Social class, Population characteristics, Socioeconomic determinants of health, Health inequality, Singapore, Population health, Health services research

## Abstract

**Background:**

Socioeconomic status (SES) is an important determinant of health, and SES data is an important confounder to control for in epidemiology and health services research. Individual level SES measures are cumbersome to collect and susceptible to biases, while area level SES measures may have insufficient granularity. The ‘Singapore Housing Index’ (SHI) is a validated, building level SES measure that bridges individual and area level measures. However, determination of the SHI has previously required periodic data purchase and manual parsing. In this study, we describe a means of SHI determination for public housing buildings with open government data, and validate this against the previous SHI determination method.

**Methods:**

Government open data sources (e.g. data.gov.sg, Singapore Land Authority OneMAP API, Urban Redevelopment Authority API) were queried using custom Python scripts. Data on residential public housing block address and composition from the HDB Property Information dataset (data.gov.sg) was matched to postal code and geographical coordinates via OneMAP API calls. The SHI was calculated from open data, and compared to the original SHI dataset that was curated from non-open data sources in 2018.

**Results:**

Ten thousand seventy-seven unique residential buildings were identified from open data. OneMAP API calls generated valid geographical coordinates for all (100%) buildings, and valid postal code for 10,012 (99.36%) buildings. There was an overlap of 10,011 buildings between the open dataset and the original SHI dataset. Intraclass correlation coefficient was 0.999 for the two sources of SHI, indicating almost perfect agreement. A Bland-Altman plot analysis identified a small number of outliers, and this revealed 5 properties that had an incorrect SHI assigned by the original dataset. Information on recently transacted property prices was also obtained for 8599 (85.3%) of buildings.

**Conclusion:**

SHI, a useful tool for health services research, can be accurately reconstructed using open datasets at no cost. This method is a convenient means for future researchers to obtain updated building-level markers of socioeconomic status for policy and research.

## Introduction

Socioeconomic status (SES) is a well-established determinant of health and is relevant to medical research and public health policy [1]. SES data is of high value to policy makers and researchers, and is frequently used to control for confounding in health services and epidemiology research [2, 3]. However, obtaining individual-level SES measures (e.g. education, income and occupation) is challenging, as they are considered sensitive information. They are subject to more stringent confidentiality protections and regulatory barriers with respect to collection and sharing of data. These difficulties often mean that individual-level SES data needs to be collected repetitively for separate studies. Direct data collection of SES markers can also be vulnerable to biases such as recall bias and social desirability bias [4].

Area-level SES measures, such as census tract and neighbourhood-level studies, have thus emerged as alternatives to individual-level measures [5]. However, area measures that cover a large heterogeneous subpopulation are vulnerable to the ecological fallacy (i.e. wrongly classifying individuals’ characteristics when using group level data). Therefore, a building-level marker of SES is a sensible compromise to bridge the limitations of area-level and individual-level SES data [6].

The Singapore Housing Index (SHI), also known as the “room-index”, is a Singapore-contextualised building-level, asset-based SES measure. It was first described by Wong et al. [7], and subsequently applied to a range of health services research. In brief, it classifies each building by the weighted average number of rooms of each unit, thereby taking a value from 1 to 7 (with larger values indicating higher SES). Housing property value provides a good surrogate measure of SES in Singapore, where the greater that 80% of the resident population dwell in public housing [8] under a tiered subsidy scheme [9], and smaller sized flats of 1–2 room size have caps on household monthly income for eligibility [10]. The SHI applies to individuals who stay in the community (i.e. not in welfare homes for the destitute, or residential care homes for the aged). The utility of the SHI in local health services research has been demonstrated - both as a primary exposure of interest, and as a confounder to control for in clinical outcomes and healthcare utilization studies [7, 11–16]. These studies have included disease-specific cohorts (head and neck cancer, breast cancer), various demographic subgroups (elderly), and both in-hospital and out-of-hospital settings. However, previous use of the SHI has required purchase of data from government agencies (e.g. Singapore Land Authority) to construct and update the index, due to urban renewal and construction. The exact methodology has varied with the availability of paid and open sources of data at the time of analysis, and has been an expensive undertaking, with recurrent expenditure for updated data. This has posed a significant barrier to using the SHI building-level SES measure for research.

We hypothesized that new open data sources would provide an alternative means of obtaining SES data. Open data is defined as structured data that is machine-readable, freely shared, as well as used and built on without restrictions [17]. It may be provided by government agencies or private bodies, and resides in the public domain. In Singapore, the majority of government open data is hosted at the National Open Data Portal (data.gov.sg) [18], which is administered by Smart Nation Singapore and has been live since 2018. Other government agencies may also provide open data on their respective websites or data portals. Updates to open data are immediately available to researchers and are free of charge, unlike periodically purchased data. They may also be accessed by interacting with the application programming interfaces (APIs) of the respective data portals, which are a software interface for data users to automatically request data from the open dataset via structured code queries.

In this paper, we describe the use of APIs and other public-domain datasets provided by the Singapore Government (via data.gov.sg) and other government sources (Singapore Land Authority, Urban Redevelopment Authority, Ministry of Health, Attorney-General’s Chambers), to reconstruct the Singapore SHI. We hope that description of these methods will improve accessibility to policy and research users. We compared the SHI obtained by this method with the original SHI dataset by Wong et al. [7], which was curated from non-open data sources in 2018.

## Methods

### Data retrieval methods

Code for data retrieval was written in Python 3.6.9, using the Google CoLaboratory environment. Calls to open data APIs were made using the *requests* library. They were formatted into the standardised JavaScript Object Notation (JSON) format using the *json* library.

The data we sought to obtain related to housing block address, postal code and housing block composition. Other additional data that was not required for SHI determination, but which we deemed germane to SES, included geographical coordinates (latitude and longitude) of the housing block, as well as recently transacted property prices.

The data source of the respective data types is detailed in Table 1 below, and are classified by whether they give information on public housing (i.e. Housing Development Board (HDB) properties), private housing, or excluded addresses (i.e. welfare homes and residential care homes). Excluded addresses are those to which the SHI does not apply, as individuals with these addresses by definition are not residing in their own property. Overall, open data sources included the HDB Property Information [19] and HDB Resale Flat Prices [20] datasets from data.gov.sg, the Singapore Land Authority (SLA) OneMAP API [21], the Urban Redevelopment Authority (URA) API [22]). Other public data sources were the Ministry of Health (MOH) HealthHub [23], and the Attorney-General’s Chambers (AGC) Singapore Statutes Online (SSO) [24].

**Table 1:** Data source for various data types

	**Public Housing**	**Private Housing**	**Excluded**
**Address**	HDB Property Information dataset	Not available	Singapore Statutes Online (Destitute Homes) HealthHub (Nursing Homes)
**Block Composition**	HDB Property Information dataset	NA	NA
**Postal Code**	SLA OneMAP API	Not available	NA
**Geographic Coordinates**	SLA OneMAP API	SLA OneMAP API	NA
**Recently Transacted Property Prices**	HDB Resale Flat Prices dataset	URA API	NA

*NA* Not applicable

The main data available in the public domain relates to public housing. We began with the HDB Property Information dataset via an API call to data.gov.sg, which contains exhaustive data on all HDB properties updated as of January 2021. This was filtered for residential properties only, with the block address and block composition (i.e. number of units of different size) available. No postal codes were available in this dataset. We hence processed the addresses into a structured text query, and made API calls to the OneMAP API to obtain the corresponding postal code and geographical coordinates.

As the HDB Property Information dataset is exhaustive and up to date, individuals whose address or postal code do not fall within it are not public housing residents. These individuals’ addresses were checked against an “Excluded Addresses Dataset” consisting of the exhaustive list of welfare homes (documented in the Destitute Persons Act [25]) and residential care homes in Singapore (documented in HealthHub [26]). An individual with an address or postal code that was in none of the above datasets was assumed to reside in private housing.

An optional step, which we did not pursue in this study, would be to verify that the given address or postal code is a residential property. This can be done via the URA API, but requires registration with a valid business email and a personalised token to access. This step is likely to be superfluous in Singapore, given that the National Registration Act [27] mandates all individuals with a registered identity card in Singapore (i.e. all residents in Singapore) to have a valid locally registered residential address.

### Calculation of the SHI

The SHI described by Wong et al. [7] classifies each building by the weighted average number of rooms of each unit, except for private housing. It takes a value from 1 to 7. Code 6 is universally assigned to private apartment housing, while code 7 is assigned to private landed properties. For public housing blocks, the weighted mean number of rooms per apartment in the building is taken, thus yielding an SHI value ranging from 1 to 6 for each block. The number of rooms in a given public housing apartment is capped at 6, including all public housing apartments larger than 5 rooms (i.e. special types such as executive condominiums, executive maisonettes, and multigeneration flats).

For the current open data formulation of the SHI, the exact composition of each public housing building was available, and hence the SHI could be directly calculated. The original formulation by Wong et al. had an algorithm for assigning SHI values where the exact composition of the block was not known, but we had no need of such an algorithm here.

The SHI for private housing described by Wong et al. used the Singapore Land Authority postal code master plan, and classified the postal codes labelled as private housing (no government subsidy) as SHI 6 (condominium apartment building) or 7 (landed residence such as bungalows or terrace houses). In the current open data formulation of the SHI, no open dataset exists that can exhaustively identify private housing, condominiums or landed residences, and hence the distinction between private housing buildings of SHI 6 and 7 could not be made. If future open data is available to make this distinction, we recommend that such data be used. As an interim measure, we recommend that all individuals whose addresses do not fall into the exhaustive HDB Property Information dataset, or the Excluded Addresses Dataset, be coded as SHI 6.

### Retrieval of other related data and SES data

Although previous work has focused on the SHI as a measure of building-level SES, we recognise that other researchers may wish to examine data on property transacted prices as a surrogate measure of the individual’s asset value, as has been done in other countries [28]. This data may be extracted from the HDB Resale Flat Prices dataset (data.gov.sg) for public housing, and the Urban Redevelopment Authority API for private housing.

We also note that geographic coordinates of a given postal code or address may be useful for researchers seeking to perform area-level aggregation. These may be queried from OneMAP.

### General overview

**Fig. 1:**
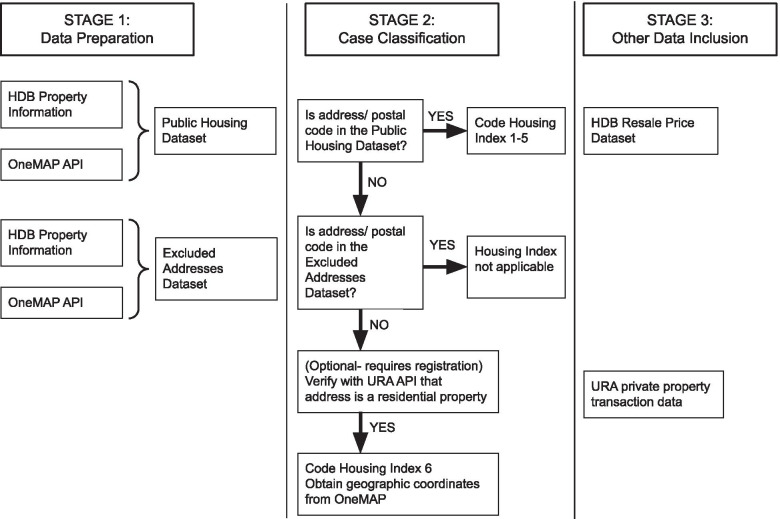
Algorithmic representation of open data workflow, depicting the stages of data preparation from open data sources, case classification process, and potential inclusion of other data

The overall workflow for open data is summarised in Fig. 1.

### Validation of open data SHI

We performed validation of the open data derived SHI dataset against the original SHI dataset by Wong et al. (data was obtained from the authors of that study). Only public housing buildings were validated, as differentiation between condominiums (SHI 6) and landed residences (SHI 7) was not possible from open data.

We assessed the overlap of properties in the two datasets by matching via postal codes and addresses. For properties that were included in both datasets, we assessed agreement between the SHI derived from the two sources by examining the intraclass correlation coefficient (ICC), and Bland-Altman plot analysis. ICC was calculated using the *pingouin* package (an additional package installed on top of base Python), and the Bland-Altman plots were generated with base Python.

We performed further validation by investigating cases where buildings could not be matched by address and postal code. These were triangulated by manual searching against two other public data sources - SingPost Find Postal Code search [29], and Google Maps [30].

## Results

### Dataset matching

Details of 21,272 HDB buildings were extracted from data.gov.sg, of which 10,077 unique buildings were labelled as residential. API calls to OneMAP were made for all residential buildings. Valid geographical coordinates were obtained for all (100%) buildings. 10,012 (99.36%) of the buildings had a valid postal code, as 65 (0.64%) returned a NIL result.

We examined the overlap between the 10,077 buildings included in the open dataset, and the original dataset by Wong et al., which had 10,012 unique HDB residential buildings. Buildings were first matched by postal code and address, and 9888 buildings were successfully matched in this fashion. They were then matched by the address only, and 123 more buildings were matched in this manner. The total number of matched buildings was thus 10,011.

**Fig. 2:**
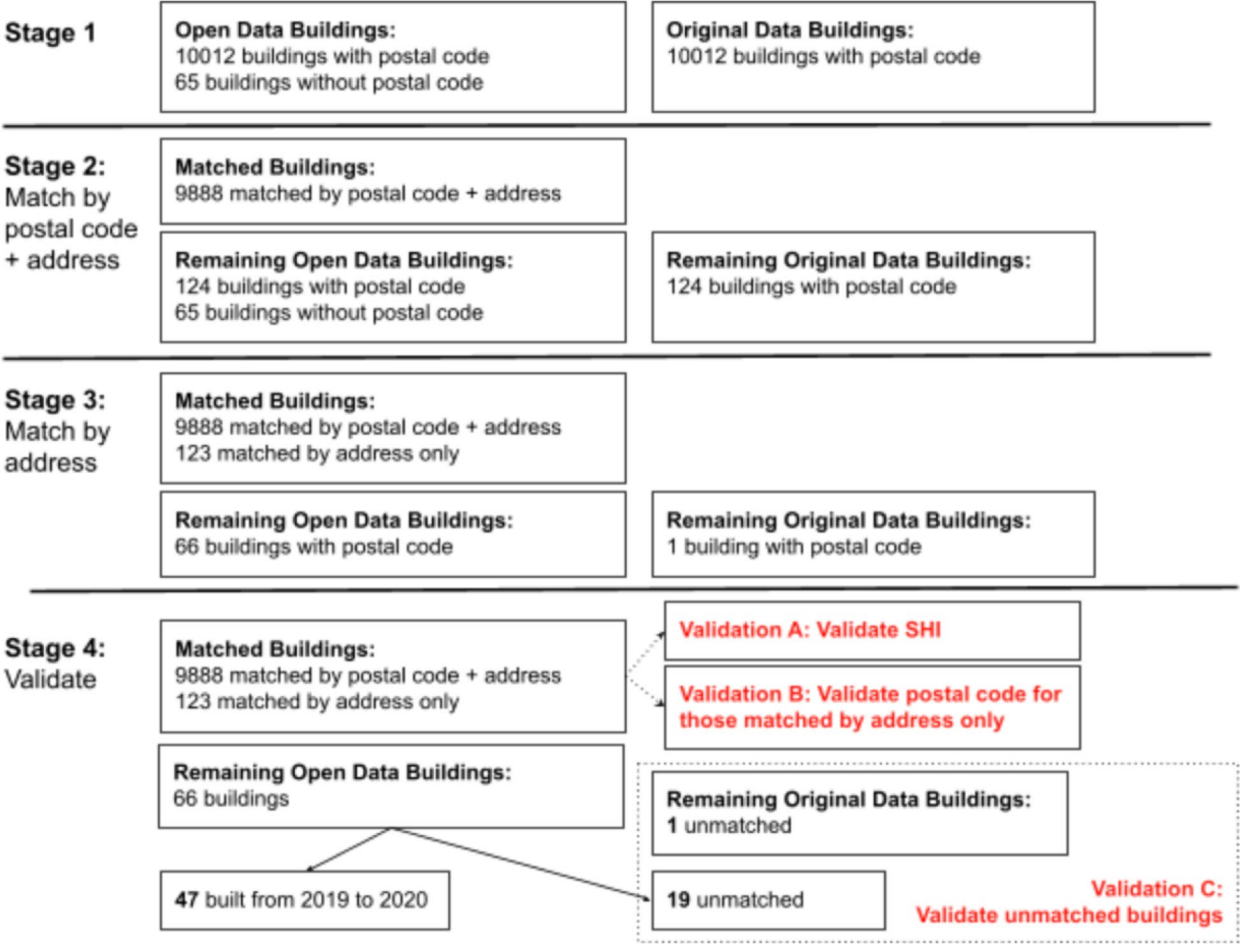
Description of the staged matching of buildings from the Original and Open datasets, with description of validation steps

A total of 67 buildings could not be matched between the two datasets. Of these buildings, 47 were HDB blocks that were newly constructed in 2019 and 2020. As the dataset by Wong et al. had only been updated in 2018, we decide not to pursue further validation for these buildings. The remaining 20 buildings could not be matched by any other means. They were not in the ‘Excluded Buildings’ dataset as well. The overall process of matching is depicted in Fig. 2.

### Dataset validation

Three types of validation were performed. SHI was compared between the original and open datasets in Validation A. Validation B examined cases where buildings could not be matched between original and open datasets via postal code, and Validation C examined buildings that could not be matched between original and open datasets at all.

#### Validation A: validation of SHI

We performed validation of the SHI for the 10,011 properties that could be matched via the combination of postal code and address, and the address alone. SHI was calculated for all properties in the open dataset. We initially mirrored the formula in Wong et.al. In doing so, we noted two potential limitations to the original calculation by Wong et al. - First, that studio flats were treated as having 3 rooms (in fact, they often only have 1 room); Second, that an uncommon type of 4 room rental flat (*“other_room_rental”*) present in 9 housing blocks had been excluded in the original calculation, resulting in some blocks having an SHI of 0.

**Fig. 3:**
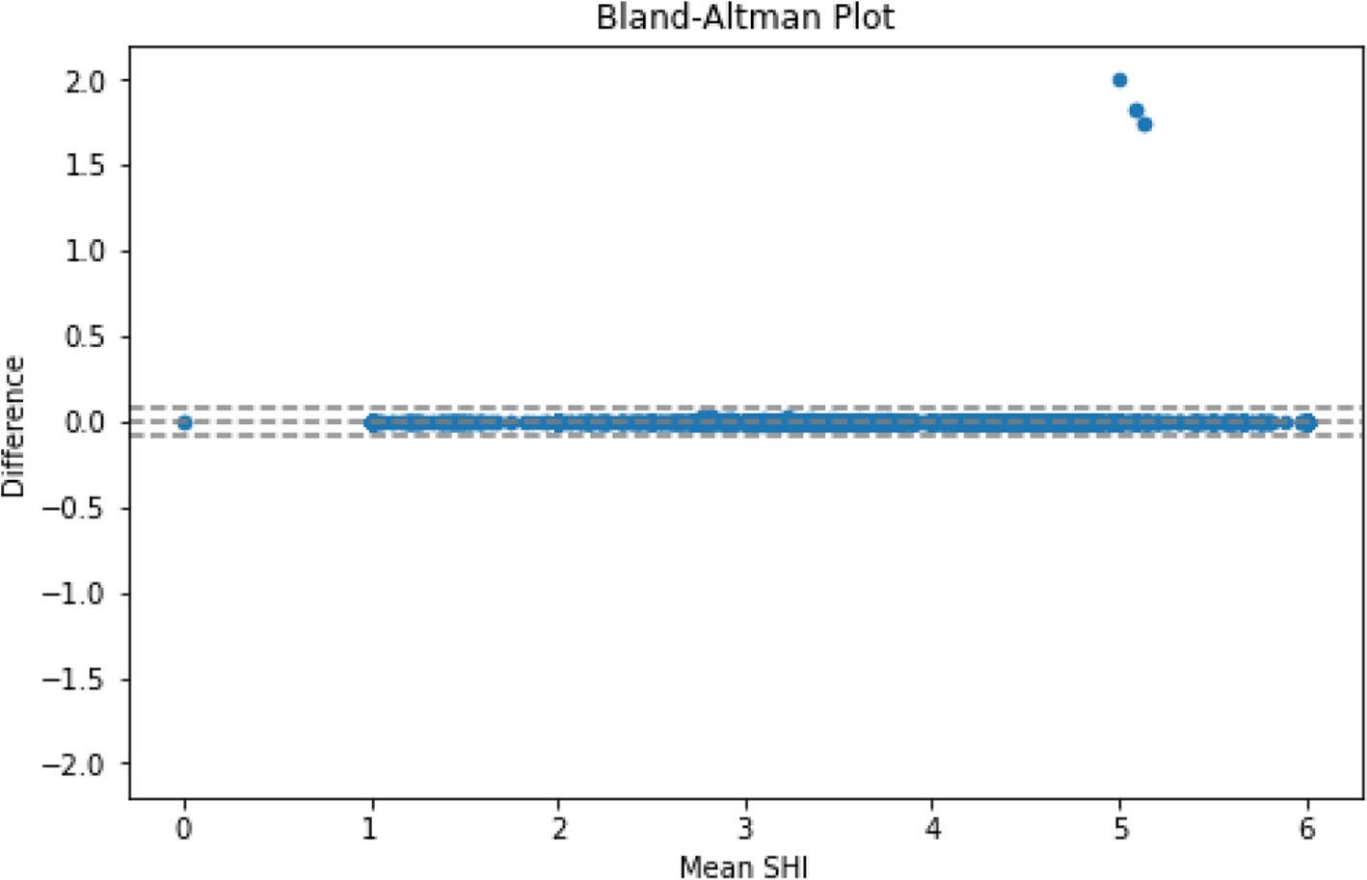
Bland-Altman Plot of SHI Difference against Mean SHI, with 2 Standard Deviation Confidence Limits

For the matched properties, we performed SHI validation by examining the intraclass correlation coefficient (ICC). This was 0.999, indicating near-perfect agreement. We also generated the Bland-Altman plot (Fig. 3), which also showed near-perfect agreement between the two sources of SHI, as evidenced by the majority of the points falling within the 2 standard deviation band for difference. However, we noted a small number of outliers, with an SHI difference of > 0.1 between original and open datasets. Manual investigation of these revealed 5 properties that had an incorrect SHI of 6 assigned by the original dataset.

In lieu of Wong’s original formula, researchers in future may consider using an updated formula with the following changes:

- Excluded addresses are assigned an SHI of 0
- Studio flats are counted as having 1 room
- Flats in the *“other_room_rental”* category are counted as having 4 rooms

#### Validation B: validation of postal codes

Of the 123 buildings matched by address only, 65 had no valid postal code returned by the OneMAP API. These buildings did have valid postal codes from SingPost and Google Maps, and we interpreted these as errors made by the OneMAP API.

Ten buildings had two postal codes available as per SingPost. For these buildings, we interpret this as both original and open datasets being correct despite the discrepancy. Forty-six buildings had postal codes that only differed by the third digit. These indicate buildings with the same address, but have different postal codes because of multiple postal delivery points [31]. For these buildings, we manually verified that the text addresses were similar, and thus interpret these situations as both original and open datasets being correct.

Finally, we noted two buildings where a postal code for a different building was returned by the OneMAP API. This was confirmed by referencing both SingPost and Google Maps. All discrepancies noted in this section were reported in writing to the Singapore Land Authority, and were forwarded to the OneMAP technical team for review.

#### Validation C: validation of unmatched buildings

Of the 20 unmatched buildings, 1 was from the original dataset, and 19 were from the open dataset. By referencing SingPost and Google Maps, we confirmed that all 20 buildings existed, with a valid postal code and address. We interpret these as unintended omissions, suggesting that the open dataset had omitted 1 building, and the original dataset had omitted 19 buildings. Of these 20 buildings, based on search engine results, all appeared to be residential addresses.

### Other SES data: recent transaction prices

**Fig. 4:**
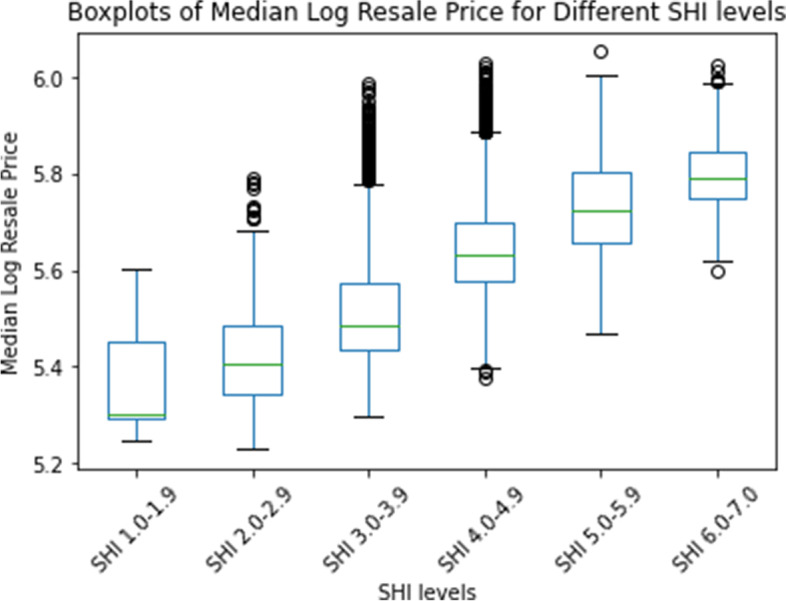
Boxplots of Median Log Resale Price at Different SHI Levels

Eight thousand five hundred ninety-nine public housing blocks had resale transaction data in the last 2 years (2019 and 2020). We matched this data to the open SHI dataset using the building address and calculated the median resale price over the 2 years. The boxplots of median log resale price, stratified by the SHI for each building, are shown in Fig. 4. There is a clear monotonically increasing relationship between the SHI of a building and the overall resale prices.

## Discussion

In this paper, we described a method of using open data to obtain the SHI of an individual based on their address or postal code. We provided example Python code in the Google CoLaboratory environment for other researchers to replicate the workflow and to demonstrate use of the respective open data APIs. These software tools are all open source and free to use. An SHI dataset created by this method can be readily linked with clinical datasets via the postal code or address, with the merged dataset subsequently utilized for data analysis.

Previous local healthcare research has successfully leveraged public data [32–35]. In describing these methods, we hope to increase awareness of the use of open data APIs, which have the advantages of convenience of use, containment of research costs, and better comprehensiveness in the case of government sourced open data. Specifically, we document in this paper how a tool used to estimate area-level SES (i.e. the SHI) can be determined using freely available open data, without the need to incur research costs as was previously necessary. We also note that usage of APIs in this context improves recency, and reduces chance of error as no human curation is required. In this study, we discovered a small number of cases where a wrong SHI had been assigned in the original dataset, presumably due to human error. Overall, we anticipate that this method described may benefit health services and epidemiology researchers.

Use of open data means that researchers are limited to the data fields provided by the source. This limitation was encountered in the current methodological description, where private residential addresses were determined through a process of elimination, as there was no existing open dataset for this. For the current SHI workflow, such assumptions are reasonable given that public and private housing in Singapore are essentially mutually exclusive, but this limitation may constrain other applications of open data locally. We note that in the current SHI application, open data reduced our ability to differentiate private residential addresses into condominium (SHI 6) and landed property (SHI 7). However, we were able to identify destitute homes, which was a limitation of the original methodology. Future researchers may consider assigning a separate code (e.g. SHI 0) to this group of individuals, or grouping them together with rental flat occupants. We were also able to identify residential nursing homes, and future researchers may wish to consider this as a separate group, especially for research involving older residents. Information on whether these are voluntary or private nursing homes is also available from the MOH HealthHub data source, if added granularity is desired.

Researchers using open data must trust the data source for veracity, completeness, and recency. This is an entirely reasonable assumption for local government sourced open data, given a stated commitment to providing timely and high-quality data [36]. Commercially sourced or other community sourced open data may not have such a commitment. In this study, we noted that the government sourced open data had valid results in the vast majority of cases, but did have a very small amount of data errors. For example, a NIL postal code was returned from the OneMAP API for some buildings. These were clarified with the data administrators of the relevant authority. As with any data source, researchers making use of open data need to perform validation checks prior to use, and we were able to resolve these cases by corroboration with other public government data sources.

In this study, we validated the SHI obtained from open data against the original SHI dataset by Wong TH et al. This showed near-perfect agreement and suggests that the open data version is practically equivalent to the manually curated version. We do however acknowledge that there are shortcomings to both methods. The current open dataset showed a small number of buildings with a wrong SHI computation by Wong et al. On the other hand, correct postal codes could not be retrieved for a small number of addresses via open data. These factors should be weighed by researchers who are contemplating either method of determining the SHI.

Other considerations for use of open data include research data governance - at present, usage of open data does not require Institutional Review Board (IRB) approval, as local IRBs do not have jurisdiction over data in the public domain. However, users of open data need to be familiar with the relevant licenses the data is provided under, and the acceptable terms of use. For example, the Singapore Open Data License [37] for data.gov.sg allows commercial and non-commercial use, but prevents users from assuming patent, trademark, or design rights.

Readers should also be aware that data in the public domain is not necessarily open data. In the current context of SHI determination, other information on property classification might be freely and publicly available on property agency websites. However, such websites are intended for human use and not for automated querying, and would generally not have APIs available. While data may still be programmatically obtained from such websites using web scraping software, this may not be the intention of the site owners and may be perceived as malicious online behaviour. Usage of web scraping tools is beyond the scope of this article, but we encourage fellow researchers to review the terms of use and the *robots.txt* file (a file describing acceptable use of automated web page retrieval for a given website) when interacting with web data sources that are not explicitly identified as open data.

The validity, strengths and limitations of SHI as a SES marker are beyond the scope of this study. The SHI can potentially be incorporated into composite indices using methods such as Principal Component Analysis. This approach of constructing building-level property-value indices as a SES marker could potentially be employed in a similar fashion outside Singapore.

## Conclusion

We developed and described a workflow for re-constructing the Singapore Housing Index using open data. This provides a means for future researchers to obtain updated building-level markers of socioeconomic status for policy and research.

## Data Availability

All data used is freely available and the code to access and manipulate the data is available at: https://colab.research.google.com/drive/1sdWtP4e-mIkDpU6pMdGrzpNvD_RJ8bBe?usp=sharing.
